# Re: Tyagi comments on “*In situ* observation of urothelial responses to NaCl-induced osmotic stress using optical coherence tomography”

**DOI:** 10.1117/1.JBO.31.3.039702

**Published:** 2026-03-02

**Authors:** Hui Wang, Hui Zhu

**Affiliations:** a Miami University, Department of Chemical and Biomedical Engineering, Oxford, Ohio, United States; b Glickman Urological and Kidney Institute, Urology Section Louis Stokes Cleveland Veterans Affairs Medical Center, Cleveland Clinic Foundation, Department of Urology, Cleveland, Ohio, United States

## Abstract

The letter responds to the comments from P. Tyagi in the same issue.

**To the Editor:**

We thank Dr. Tyagi for his thoughtful commentary on our paper, “*In situ* observation of urothelial responses to NaCl-induced osmotic stress using optical coherence tomography.”^1^ We agree that the protective role of bladder perfusion is a fundamental principle in urological physiology. Our study employed an *ex vivo* model not to contradict this principle, but to remove these systemic protections deliberately, allowing us to isolate and directly visualize the inherent, biophysical responses of the urothelial tissue itself to a defined osmotic stimulus using optical coherence tomography (OCT). The primary contribution we emphasize is methodological: demonstrating OCT’s unique capability for real-time, micrometer-scale imaging of dynamic structural changes in the urothelium under stress. We envision that this technology has potential for future development as an *in vivo* imaging tool to advance the study of urothelial function.

We explicitly acknowledged the limitation of the *ex vivo* model in our manuscript, noting that “it does not fully replicate the complex physiological conditions present *in vivo*, such as blood flow and hormonal regulation,” and our goal was not to reproduce the healthy, well-perfused bladder but to isolate the urothelium and study its intrinsic responses with OCT. This approach is relevant for diseases where these protective systems are degraded. Although an *ex vivo* system is qualitatively different from the integrated *in vivo* state, lacking blood flow, neural input, and hormonal regulation, this simplification enables the clean interrogation of how osmotic gradients directly affect urothelial structure. The clinical relevance of studying a vulnerable urothelium is underscored by the literature. For instance, Özçağlayan et al.^2^ reported that “arterial blood flow of bladder was lower in patients with BPS compared with the control group” and that “the decrease in the vascular supply of bladder might be one of the related factors for the BPS etiology.” A well-documented complication of pelvic radiation for bladder and prostate cancers is overactive bladder (OAB). The radiation causes diminished blood flow (ischemia) to the bladder, leading to radiation cystitis and subsequent OAB symptoms. Thus, although our model does not recreate the full disease pathology, it establishes a controlled platform to investigate a key component: how the absence of perfusion might influence urothelial susceptibility to osmotic stress. Our goal was to establish a baseline understanding of the urothelium’s physical limits, not to model the diseases in their entirety.

Tyagi cites Morizawa et al.^4^ to argue that our “alleged role of osmotic gradient is refuted” and that water transport at the cellular level is unidirectional and purely passive. However, a critical re-examination of their published data challenges this interpretation and aligns with a role for solute-driven transport. Their key finding is that intravesical volume decreased significantly more with isotonic saline (∼21%) than with isotonic 5% glucose (∼6%) over 3 h. Notably, the osmolality of the 5% glucose solution increased substantially to 345  mOsm/L. This demonstrates that water absorption occurred alongside, and likely resulted in, a marked increase in luminal osmolality. The authors attribute the greater absorption with saline to sodium-dependent upregulation of AQP-2 and specific claudins, concluding the bladder “absorbs water using upregulated AQPs if filled with fluid containing sodium.” Therefore, Morizawa et al. do not refute osmotic or solute-driven water movement; their work highlights that sodium uniquely enhances water absorption, likely by increasing tissue permeability. This conclusion is entirely compatible with an osmotic framework where solutes (such as NaCl) modulate the driving force and/or the tissue’s conductive properties.

We acknowledge that our *ex vivo* model cannot delineate the specific molecular mechanisms (e.g., AQP-2 upregulation) reported in *in vivo* studies like Morizawa’s. Our contribution is complementary: we provide direct, real-time visualization of the immediate structural consequences (swelling, shrinkage, altered light scattering) that occur as water moves in response to an applied osmotic gradient. These structural events, observable on a timescale of seconds, are the physical manifestations of the net water movement measured over hours in studies similar to Morizawa’s. Our observations are therefore fully consistent with an osmotic model where sodium modulates permeability, and they provide a novel, complementary morphological readout of the process. Moreover, neither Morizawa et al.^5^ nor the clinical TVR observations cited by Tyagi demonstrate an intrinsically “unidirectional” water transport mechanism at the microscopic tissue scale. AQP-mediated and paracellular water movements are physically bidirectional. Our OCT results add the missing micro-scale, dynamic view of these rapid structural events, which is not captured by slower, macro-scale volume measurements.

Regarding the use of NaCl and higher osmolarities, our primary goal was methodological: to apply a well-defined and controllable osmotic stimulus to systematically probe the intrinsic structural response of the urothelium. OCT imaging revealed the onset of urothelium damage at 1.03  Osm/L, whereas subtle damage was initiated at 0.69  Osm/L. Both concentrations are within a physiologically plausible range for human urine, which can reach ∼1200  mOsm/kg $H_{2}O$ (∼1.2  Osm/L) during antidiuresis.^6^ This strategy allowed us to systematically map the progression from initial, subtle injury to more severe structural disruption, thereby demonstrating both the sensitivity of our OCT-based assay and the continuum of osmotic injury. Using NaCl alone as the sole osmotic probe was deliberate, enabling us to isolate the effects of sodium-driven osmotic stress and to connect our findings with prior work showing that NaCl, but not urea or glucose, robustly upregulates AQP3 and increases water flux across the urothelium under an imposed gradient.^7^ This is also conceptually aligned with clinical observations, such as those by Matsuo et al.^8^ They found that dietary salt restriction improved OAB symptoms, linking systemic sodium balance to bladder sensory function.

In conclusion, our work establishes OCT as a powerful tool for imaging real-time urothelial biophysics. The *ex vivo* model is a necessary first-step platform to define these fundamental responses in isolation. The structural vulnerability we document under osmotic stress generates the hypothesis that a similarly vulnerable urothelium *in vivo*, perhaps due to reduced perfusion as seen in IC/BPS^2^ could be sensitized to normal urinary solutes, contributing to symptomatology. This hypothesis requires testing in more complex, integrated models. Our main contribution lies in providing the high-resolution imaging methodology to make such future investigations, including *in vivo* studies, possible.
